# Alleviation of migraine symptoms by application of repetitive peripheral magnetic stimulation to myofascial trigger points of neck and shoulder muscles – A randomized trial

**DOI:** 10.1038/s41598-020-62701-9

**Published:** 2020-04-06

**Authors:** Tabea Renner, Nico Sollmann, Florian Heinen, Lucia Albers, Florian Trepte-Freisleder, Birgit Klose, Helene König, Sandro M. Krieg, Michaela V. Bonfert, Mirjam N. Landgraf

**Affiliations:** 1 Department of Pediatric Neurology and Developmental Medicine and LMU Center for Children with Medical Complexity, Dr. von Hauner Children’s Hospital, LMU – University Hospital, Ludwig-Maximilians-Universität, Munich, Germany; 2 Department of Diagnostic and Interventional Neuroradiology, Klinikum rechts der Isar, Technische Universität München, Munich, Germany; 3 TUM-Neuroimaging Center, Klinikum rechts der Isar, Technische Universität München, Munich, Germany; 4 Department of Neurosurgery, Klinikum rechts der Isar, Technische Universität München, Munich, Germany

**Keywords:** Headache, Migraine, Paediatric neurological disorders

## Abstract

Migraine is a burdensome disease with an especially high prevalence in women between the age of 15 and 49 years. Non-pharmacological, non-invasive therapeutic methods to control symptoms are increasingly in demand to complement a multimodal intervention approach in migraine. Thirty-seven subjects (age: 25.0 ± 4.1 years; 36 females) diagnosed with high-frequency episodic migraine who presented at least one active myofascial trigger point (mTrP) in the trapezius muscles and at least one latent mTrP in the deltoid muscles bilaterally prospectively underwent six sessions of repetitive peripheral magnetic stimulation (rPMS) over two weeks. Patients were randomly assigned to receive rPMS applied to the mTrPs of the trapezius (n = 19) or deltoid muscles (n = 18). Whereas the trapezius muscle is supposed to be part of the trigemino-cervical complex (TCC) and, thus, involved in the pathophysiology of migraine, the deltoid muscle was not expected to interfere with the TCC and was therefore chosen as a control stimulation site. The headache calendar of the German Migraine and Headache Society (DMKG) as well as the Migraine Disability Assessment (MIDAS) questionnaire were used to evaluate stimulation-related effects. Frequency of headache days decreased significantly in both the trapezius and the deltoid group after six sessions of rPMS (trapezius group: p = 0.005; deltoid group: p = 0.003). The MIDAS score decreased significantly from 29 to 13 points (p = 0.0004) in the trapezius and from 31 to 15 points (p = 0.002) in the deltoid group. Thus, rPMS applied to mTrPs of neck and shoulder muscles offers a promising approach to alleviate headache frequency and symptom burden. Future clinical trials are needed to examine more profoundly these effects, preferably using a sham-controlled setting.

## Introduction

According to the Global Burden of Disease Study more than one billion people were suffering from migraine in 2016, making migraine one of the most prevalent neurological disorders worldwide^1^. Especially women between the age of 15 and 49 years are concerned – in this group migraine is also the first cause of disability worldwide^1–4^. Further, migraineurs show a considerably reduced health-related quality of life (QoL) and considerable loss in work productivity^5–7^. The high impact of migraine on QoL is reflected by percentages as high as 85% of those affected who feel helpless, depressed or not understood, with 83% reporting sleeping difficulties and 55% being afraid of the next migraine attack^8^. However, this frequent and debilitating headache disorder is still under-diagnosed and often inadequately treated^9^.

Despite the high global prevalence of migraine and the considerable worsening of QoL among affected subjects, the complex pathophysiology of migraine is not completely understood^10,11^. Over the recent years, the neck and shoulder region of migraineurs is more and more focused on in the field of migraine research. Specifically, neck pain may play an important role as a trigger or premonitory symptom or part of migraine attacks, with muscular pain and generalized hyperalgesia in the neck and shoulder region being more frequent in subjects with migraine than in healthy controls^12–16^. The interrelation of neck pain and migraine might be explained by the concept of the trigemino-cervical complex (TCC), which suggests that peripheral sensitization of trigemino-cervical neurons may influence central nociception by a potential convergence of cervical and dural nociceptive afferents in the caudal nuclei of the trigeminal nerves in the brain stem^17–19^. Myofascial trigger points (mTrPs) in migraineurs could be regarded key components related to muscular pain and hyperalgesia as well as headache symptoms, and mTrPs have shown to respond positively to local treatment and, thus, could be promising sites for targeted intervention^20–25^. Hence, mTrPs in the neck and shoulder region seem to be a gateway to enter and influence the TCC with the aim of achieving symptom alleviation in migraineurs^20^.

Central modulation in migraineurs can be reached by different invasive and non-invasive techniques^26–29^. Promising examples include transcranial magnetic stimulation (TMS)^30–33^, transcranial direct current stimulation (tDCS)^34–36^, supra-orbital nerve stimulation (SONS)^37–39^, and vagus nerve stimulation (VNS)^40–43^. In addition, repetitive peripheral magnetic stimulation (rPMS) has been introduced recently as a novel method to stimulate the upper trapezius muscles in subjects with migraine, with its application being feasible, well-tolerated, and mostly free of any adverse effects among migraineurs^44^. Moreover, migraine frequency decreased substantially according to three-month follow-up after rPMS to trapezius muscles as assessed by the Migraine Disability Assessment (MIDAS) questionnaire^44^. Of note, the local pressure pain threshold (PPT) of the trapezius muscles was steadily increasing during the course of the six sessions of rPMS applied in this previous study, indicating local muscular effects besides alleviation of headache symptoms^44^. In another trial rPMS was administered either to the trapezius muscle (considered part of the TCC) or the deltoid muscle (considered not to be part of the TCC) to evaluate local effects and differences depending on the muscle stimulated^45^. Despite using the same stimulation protocol for both the trapezius and deltoid muscles, the trapezius muscles showed considerably higher PPTs than the deltoid muscles according to post-interventional examination, which was found regardless of the stimulated muscle (rPMS to the trapezius but also the deltoid muscles influenced the PPTs in trapezius muscles)^45^.

These previous results imply that rPMS could alleviate muscular sensitivity at the stimulated area as well as headache symptoms within the context of the TCC, making rPMS a promising neuromodulatory technique^44^. To date, rPMS offers an auspicious non-invasive neuromodulatory approach that can directly intervene at peripheral muscular structures in the neck and shoulder area whilst potentially using the TCC as a gateway to modulate central nociception simultaneously. However, a closer look at the clinical outcome following rPMS applied to muscles inside and outside of the TCC in migraineurs is still needed to evaluate in detail the efficacy and specificity of this approach within the concept of the TCC.

Against this background, the aim of this study is to systematically investigate potential central effects of rPMS applied to either the trapezius or the deltoid muscles. We hypothesize a stronger alleviation of migraine symptoms when rPMS is applied to the trapezius muscles when compared to the deltoid muscles.

## Results

### Demographics and baseline characteristics

Table 1 gives an overview about demographics and baseline characteristics of the 37 participants included in this study. Thirty-six of them were female, one was male. The mean age was 25.0 ± 4.1 years (range: 19–35 years). The included subjects were randomly assigned to the trapezius group (n = 19) or the deltoid group (n = 18). No significant differences were found between subjects of the trapezius group compared to subjects of the deltoid group regarding demographics or items of the headache diary of the German Migraine andHeadache Society (DMKG) or the MIDAS questionnaire (p > 0.05). No dropouts were registered.

**Table 1:** Demographics and baseline characteristics according to the headache diary of the German Migraine and Headache Society (DMKG) and Migraine Disability Assessment (MIDAS) questionnaire.

	Trapezius group N = 19	Deltoid group N = 18	p*
	Median (range) or % (N)		
**Subject characteristics**			
Age (in years)	25 (19–35)	24.5 (19–32)	0.702
Female sex	100.0 (19)	94.4 (17)	0.978
**Headache diary of the DMKG (assessed daily over the course of 90 days before and after intervention)**			
Number of days with headache	23 (17–37)	20 (15–40)	0.057
Cumulative headache duration (hours)	194 (78–429)	121 (60–482)	0.448
Duration per headache attack (hours)	6.8 (4.0–14.8)	6.1 (3.3–19.3)	0.988
Average headache intensity (according to VAS)	5.3 (3.5–6.9)	5.2 (3.9–6.5)	0.727
Vomiting (incidences per 90 days)	0 (0–4)	0 (0–9)	0.521
Nausea (incidences per 90 days)	7 (0–25)	5 (0–16)	0.344
Medication (intake per 90 days)	12 (0–29)	11 (3–27)	0.927
**MIDAS questionnaire (assessed for the 90 days before and after intervention)**			
Missing school/work (days)	1 (0–5)	1 (0–12)	0.405
Productivity at school/work reduced by half (days)	10 (2–20)	7.5 (3–23)	0.247
Could not do household work (days)	5 (0–11)	4.5 (0–18)	0.903
Household work productivity reduced by half (days)	5 (0–15)	6 (0–14)	0.843
Missing family, social, or leisure activities (days)	3 (0–10)	4.5 (0–17)	0.375

*Wilcoxon signed-rank test or Chi-squared test.

### Pre- vs. post-interventional results according to the DMKG headache diary and the MIDAS questionnaire

**Figure 1:**
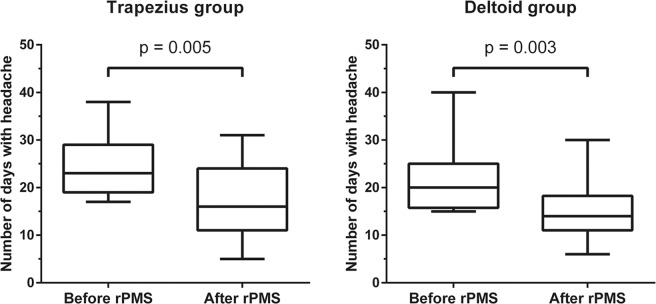
Number of days with headache. The box plots depict the number of days with headache according to evaluation by the headache diary of the German Migraine and Headache Society (DMKG), which was carried out before and after the two-week interval of repetitive peripheral magnetic stimulation (rPMS). Median values with 25% and 75% percentiles and minimum and maximum whiskers are shown separately for the trapezius group and deltoid group. There was a statistically significant difference between the pre and post-interventional assessments in both groups (trapezius group: p = 0.005, deltoid group: p = 0.003).

Table 2 gives an overview about the pre- and post-interventional status according to the DMKG headache diary and the MIDAS questionnaire for both groups. The headache frequency per 90 days significantly decreased in the trapezius group from 23 to 16 days (p = 0.005, relative reduction −34.8%) and in the deltoid group from 20 to 14 days (p = 0.003, relative reduction −32.5%; Fig. 1). Regarding the cumulative duration of headache attacks there was a tendency towards declining values (trapezius group: p = 0.068, relative reduction −23.2%; deltoid group: p = 0.076, relative reduction −37.2%).

**Table 2:** Pre- versus post-interventional results according to the headache diary of the German Migraine and Headache Society (DMKG) and Migraine Disability Assessment (MIDAS) questionnaire.

	Trapezius group N = 19		p*	Deltoid group N = 18		p*
	Pre-stimulation	Post-stimulation		Pre-stimulation	Post-stimulation	
	Median (range)			Median (range)		
**Headache diary of the DMKG (assessed daily over the course of 90 days before and after intervention)**						
Number of days with headache	23 (17–37)	16 (5–31)	**0.005**	20 (15–40)	14 (6–30)	**0.003**
Cumulative headache duration (hours)	194 (78–429)	146.5 (40–336)	0.068	121 (60–482)	97.5 (19–420)	0.076
Duration per headache attack (hours)	6.8 (4.0–14.8)	7.8 (3.6–16.9)	0.606	6.1 (3.3–19.3)	6.9 (2.8–17.5)	0.704
Average headache intensity (according to VAS)	5.3 (3.5–6.9)	5.9 (4.3–7.9)	0.161	5.2 (3.9–6.5)	5.3 (3.3–6.7)	0.584
Vomiting (incidences per 90 days)	0 (0–4)	0 (0–3)	0.523	0 (0–9)	0 (0–2)	0.819
Nausea (incidences per 90 days)	7 (0–25)	4 (0–29)	0.138	5 (0–16)	3.5 (0–17)	0.666
Medication (intake per 90 days)	12 (0–29)	9 (0–27)	0.254	11 (3–27)	9 (2–17)	0.302
**MIDAS questionnaire (assessed for the 90 days before and after intervention)**						
Missing school/work (days)	1 (0–5)	1 (0–5)	0.914	1 (0–12)	1 (0–6)	0.630
Productivity at school/work reduced by half (days)	10 (2–20)	4 (0–10)	**0.001**	7.5 (3–23)	4 (0–12)	**0.005**
Could not do household work (days)	5 (0–11)	2 (0–15)	0.095	4.5 (0–18)	2 (0–12)	0.160
Household work productivity reduced by half (days)	5 (0–15)	2 (0–7)	**0.002**	6 (0–14)	3 (0–11)	0.077
Missing family, social, or leisure activities (days)	3 (0–10)	2 (0–10)	0.324	4.5 (0–17)	2.5 (0–12)	0.245

*Wilcoxon signed-rank test. P-values printed in bold are statistically significant after correction for multiple testing using the Benjamini-Hochberg procedure with a false discovery rate (FDR) of 10%.

The MIDAS score decreased from 29 to 13 points (p = 0.0004) in the trapezius group and from 31 to 15 points in the deltoid group (p = 0.002). In both groups, the median MIDAS score changed from “severe impairment” to “moderate impairment” (trapezius group: p = 0.0005; deltoid group: p = 0.009). Overall, the MIDAS score improved significantly after rPMS (p = 0.001), considering all four MIDAS subgroups with the 37 study participants. After intervention, the productivity at school/work showed to be less affected by headache events (trapezius group: p = 0.001, relative reduction −40.0%; deltoid group: p = 0.005, relative reduction −53.3%) than prior to intervention. Also, productivity in household improved significantly after rPMS in the trapezius group (p = 0.002, relative reduction −60.0%), but not in the deltoid group.

Table 3 depicts the intervention effects for each variable and compares the effects between the trapezius und deltoid group, indicating no statistically significant differences (p > 0.05). Furthermore, we conducted a sensitivity analysis excluding the single man without any relevant changes regarding the results (Supplementary Table 1).

**Table 3:** Differences between pre- and post-interventional results according to the headache diary of the German Migraine and Headache Society (DMKG) and Migraine Disability Assessment (MIDAS) questionnaire.

	Trapezius group N = 19	Deltoid group N = 18	p*
	Median (range)		
**Headache diary of the DMKG (assessed daily over the course of 90 days before and after intervention)**			
Frequency in 3 months	8 (−9–23)	6.5 (−6–12)	0.647
Cumulative duration	45 (−69–228)	45.05 (−13–138)	0.486
Average duration	−0.1 (−6.6–4.8)	0.4 (−2.1–7.3)	0.214
Average intensity	−0.2 (−3.0–0.6)	0.1 (−1.1–2.4)	0.070
Vomiting	0 (0–2)	0 (−2–7)	0.207
Nausea	4 (−15–11)	0 (−6–9)	0.092
Frequency of use of medication	2 (−8–11)	2.5 (−12–11)	0.689
**MIDAS questionnaire (assessed for the 90 days before and after intervention)**			
Missing school/work	0 (−5–4)	0 (−3–7)	0.533
Productivity at school/work reduced by half	4 (−1–18)	4 (−1–11)	0.541
Could not do household work	1 (−5–9)	2 (−8–14)	0.583
Household work productivity reduced by half	3 (−3–14)	3 (−11–12)	0.938
Missing family, social, or leisure activities	1 (−4–4)	1.5 (−5–11)	0.427

*Wilcoxon signed-rank test.

## Discussion

This study evaluated the central effects of rPMS applied to trapezius or deltoid muscles in young adults suffering from high-frequency episodic migraine with a focus on possible differences in stimulation effects between subjects stimulated on either of these muscles. Our main findings were that days suffering from headache substantially decreased in both groups, whereas headache intensity and duration per attack did not significantly change when comparing the pre- to the post-interventional status (Tables 2 and 3; Fig. 1). Moreover, the MIDAS score, which is a measurement for the impairment in daily life due to migraine, considerably improved in both groups, with the productivity at school/work being less constrained in both groups and productivity at household being less impaired in the trapezius group after rPMS (Tables 2 and 3).

**Figure 2:**
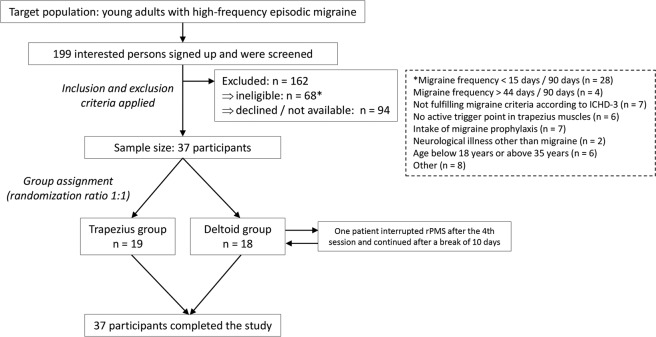
Study design and enrollment. This flow chart provides an overview of the study design, its inclusion and exclusion criteria, and group assignments. Overall, 199 subjects were screened, with a final sample size of 37 participants undergoing repetitive peripheral magnetic stimulation (rPMS) after consideration of the study’s inclusion and exclusion criteria. No dropouts were registered.

In general, alterations in neck and shoulder muscles like mTrPs as well as musculoskeletal dysfunction are supposed to play an essential role in the pathophysiology of migraine^20,46,47^. According to the concept of the TCC, peripheral sensitization and central convergence of cervical and meningeal nociceptive afferents in the brain stem could explain the important correlation of neck pain and migraine^17–19^. To date, rPMS seems to be a promising non-pharmacological, non-invasive approach that allows modulation of peripheral as well as central migraine-related symptoms via peripheral inflow, which is most likely taking effect on the basis of the TCC^44^. In a pilot study, rPMS was applied to the trapezius muscles of young migraineurs, leading to a decrease of migraine attacks and intensity of headache^44^. Furthermore, in another study local effects of rPMS on the trapezius muscles, supposed to be part of the TCC, and deltoid muscles, supposed not to be part of the TCC, were described by pre- and post-interventional measurements of the local PPT^45^. In detail, depending on the examined muscles the increase of PPTs differed significantly (subjects with stimulation of trapezius muscles: p = 0.021; subjects with stimulation of deltoid muscles: p = 0.080)^45^. Despite these promising first results focusing on the peripheral part of the TCC, further insights into rPMS and its central effects for intervention in migraine are lacking. Comparing rPMS to the latest investigations of other available neuromodulative techniques for intervention in migraine, the reduction of migraine attacks and days suffering from headache are generally in a comparable range^33,36,39,43^. In the ESPOUSE study, single-pulse TMS was applied to the occiput by the study participants twice a day during three months as a prophylactic treatment and also as an acute intervention during any migraine attack occurring in this period of time^33^. Application of single-pulse TMS led to an average reduction of 2.8 days with headache per month^33^. In comparison, tDCS applied to the area corresponding to the M1 in the dominant hemisphere ten times during three to five weeks led to a decrease of 3.0 days with headache per month^36^. Furthermore, SONS was applied to the center of the forehead by participants once a day over three months, being capable of reducing headache frequency by 2.8 days per month^39^. Participants deploying VNS three times a day for twelve weeks experienced a decline of 2.3 days with headache per month^43^. In the present study, rPMS applied to the trapezius muscle was able to decrease headache frequency by 2.7 days per month and by 2.2 days per month when applied to the deltoid muscle, respectively. It has to be noticed that rPMS, in comparison to other methods, is usually well tolerated, painless, and non-invasive^44^. Changes in the MIDAS score were only examined in a recent TMS study with a reduction of 3 points according to post-interventional evaluation^48^. In comparison, rPMS led to a clearer decrease depending on the stimulated muscle, which probably points to a considerably higher improvement in the QoL. In this context, the side effects of rPMS tend to be less severe than those reported for VNS and SONS and, consequently, might result in higher acceptance of rPMS and better satisfaction^39,43,45^.

Moreover, rPMS offers the possibility of simultaneously improving local hyperalgesia in neck and shoulder muscles of migraineurs by increasing the PPT of mTrPs in the trapezius muscles by direct or indirect stimulation^45^. Thus, on the one hand, rPMS has a substantial positive effect on musculature^44,45^. On the other hand, the peripheral modulation of the TCC via stimulation of the trapezius muscle could lead to a central modulation of nociceptive afferents in the brain stem^17,44,45^. This means that rPMS, although delivered peripherally at the muscle level, is able to influence central mechanisms that play a role in migraine pathophysiology and, hence, could improve migraine symptoms and frequency. Of note, a comparable effect occurs when rPMS is applied to the deltoid muscle in migraineurs. As the deltoid muscle is not expected to be part of the TCC, we initially hypothesized a less pronounced effect on migraine-related symptoms in the deltoid group – supported by the results of the previous publication, suggesting a more intense peripheral effect of rPMS on the trapezius muscles when compared to the deltoid muscles^45^. The positive central effect after stimulation of the deltoid muscle might be explained by the uplifting movement of the shoulder, which is provoked by rPMS on the deltoid muscle and which might indirectly active the trapezius muscle^45^. This would mean that stimulation of muscles outside the TCC that are, however, linked to the trapezius muscle might potentially allow an interaction with the central elements of the TCC in the brain stem.

Regarding the impact of rPMS among migraineurs, we have to acknowledge the important role placebo effects may play as they could generally influence the participants’ outcome considerably regarding treatment effects. In principal, the more complex a treatment is, the bigger potential placebo effects seem to be^49,50^. Especially procedures delivered by medical devices seem to entail more distinct placebo effects than oral pharmacological treatments^49^. Positive response expectancies might also intensify the placebo effect in analgesia^51^. Considering the placebo effect in migraine, there is a mean recovery rate of 40.7% in control groups according to an analysis of systematic reviews of pharmacological and non-pharmacological treatments in migraine^52^. In total, we cannot estimate the influence of a potential placebo effect as long as randomized controlled studies on rPMS are mostly lacking. Thus, a sham-controlled study is needed to explore the extent of potential placebo effects. A sham-controlled study setup could be feasible by using a dedicated sham coil that is surrounded by an isolating shell to interrupt the electric field induced by the coil, thus avoiding real stimulation as suggested for TMS applications, for instance^53^. This would mean that during its use the device’s typical noise is audible without the electric field passing the plastic tube and the musculature is, however, not stimulated^53^. Moreover, a closer look at the deltoid muscle is needed to explain the influence and effect of its stimulation regarding central effects of rPMS. In this context, recent literature on electrical stimulation of skin afferents by a wearable device applied between the bellies of the deltoid and triceps muscles supports our observations^54,55^. On behalf of this device acute migraine attacks were effectively controlled, but preventive data have not become available so far. The concept of conditioned pain modulation could be the basis for those positive effects in acute migraine treatment as well as for our observations in the deltoid group^54^. Further, assessing the effects of a novel method like rPMS on advanced migraine markers evaluated by emerging technologies, i.e. the expression of specific neurosteroid patterns or facial electronic thermography, may track progress in the understanding of distinct neuromodulatory mechanisms^56–60^.

Due to the small sample size in each group and the inclusion of young patients suffering from high-frequency episodic migraine, the results are not to be generalized to other groups of migraine patients. Moreover, the female predominance of this cohort has to be considered. However, females usually outnumber male patients in migraine trials, including studies of the other neuromodulatory approaches^61–63^. On the one hand, this fact corresponds to the overall higher prevalence of migraine in women in epidemiological studies^4,64,65^. On the other hand, this ratio tends to be even more pronounced in treatment trials – an observation not extensively studied so far.

In conclusion, this study examined central effects of rPMS when applied to mTrPs of the trapezius muscles, considered part of the TCC, and of the deltoid muscles not being supposed to be part of the TCC in young adults with high-frequency episodic migraine. After six sessions of rPMS, suffering from headache decreased substantially in both the trapezius and the deltoid group. Consequently, rPMS offers a promising tool to intervene at muscular structures in migraineurs with both central, but also peripheral effects. Further clinical studies are needed to examine more profoundly the impact of a possible placebo effect, preferably using a sham-controlled setting.

## Materials and Methods

### Ethics and study enrollment

The institutional review boards of both universities of Munich (TUM and LMU) approved the study protocol. The study was conducted in accordance with the Declaration of Helsinki. Written informed consent was obtained from all enrolled subjects. The study was registered with the German Clinical Trials Register (clinical trial registration number: DRKS00019870, 15/11/2019).

The following criteria needed to be met for inclusion: (1) age between 18 and 35 years, (2) migraine (according to the German version of the headache questionnaire modified according to the International Classification of Headache Disorders [ICHD], 3^rd^ edition^66–68^), (3) a frequency of 15 to 44 days of headache during the 90 days prior to the first rPMS intervention (verified by the headache diary of the DMKG), (4) at least one active mTrP in one of the upper trapezius muscles (identified by a physiotherapist specialized in manual palpation of mTrPs), (5) no metallic implants (e.g. pacemaker, cochlear implants), and (6) written informed consent. The following criteria were defined as exclusion criteria: (1) any neurological illnesses except for migraine, (2) intake of any medication for migraine prophylaxis, (3) any changes in hormonal contraception during rPMS or 90 days before and after rPMS, and (4) pregnancy.

Recruitment of participants was achieved via announcements in the hospitals and local libraries of the two universities of Munich. Overall, 37 subjects (mean age: 25.0 ± 4.1 years, age range: 19–35 years, 36 females) who fulfilled the inclusion criteria were enrolled (Fig. 2). Sample size estimation for the present trial was based on a previous pilot study evaluating the feasibility of rPMS to the trapezius muscles in migraineurs, reporting an average reduction of headache frequency of 33% (SD = 33)^44^. To achieve a similar effect considering statistical power of 90% and an alpha error of 5%, a sample size of 18 subjects per group would be needed. The cohort considered in the present trial has been assessed in an earlier publication for other objectives, focusing on the methodological setup presentation of rPMS and evaluation of local muscular effects of rPMS^45^.

### Study design and setup

**Figure 3:**
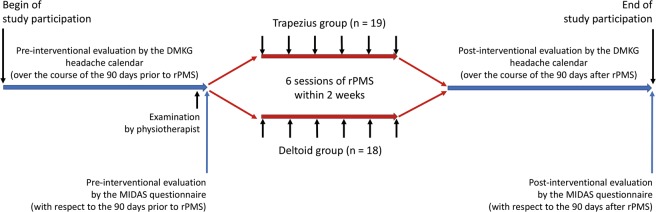
Timeline of study participation. This flow chart depicts the steps of the study in a chronological order, consisting of pre- and post-interventional assessments using the headache diary of the German Migraine and Headache Society (DMKG) and Migraine Disability Assessment (MIDAS) questionnaire. These assessments were grouped around a two-week interval of repetitive peripheral magnetic stimulation (rPMS) that was subdivided into six single sessions. Stimulation by rPMS was applied to either myofascial trigger points (mTrPs) of the trapezius muscles (trapezius group) or deltoid muscles (deltoid group). Determination of the presence and location of mTrPs was done by a physiotherapist.

We chose a monocentric, prospective, randomized study design to systematically investigate the mid-term effects of rPMS on migraine when applied to skeletal musculature (Figs. 1 and 2). The enrollment phase was between August 2016 and April 2018. The pre- and post-interventional evaluation periods lasted for 90 days each and surrounded a two-week rPMS intervention phase; thus, the complete study participation covered almost seven months (Fig. 3).

The 37 enrolled subjects were randomized into two groups with a randomization ratio of 1:1. One group was supposed to receive rPMS bilaterally on the trapezius muscles (trapezius group: n = 19), the other group on the deltoid muscles (deltoid group: n = 18). Block randomization was achieved by drawing notes labeled with one of the group assignments from a sealed envelope, which was performed by a person other than the investigator conducting rPMS. The envelope contained the same number of notes for the trapezius group and deltoid group (n = 18 each). Consideration of an additional subject in the trapezius group (n = 19) was due to initial loss of a subject during post-interventional evaluation; however, this subject was reachable again later and provided completed evaluation.

In the course of the study, each subject underwent six sessions of rPMS on the designated muscles during two consecutive weeks in regular intervals (e.g., Monday/Wednesday/Friday or Tuesday/Thursday/Saturday). The right- and left-sided trapezius or deltoid muscles, depending on group assignment, were consecutively stimulated in each session, with the starting side of the first session being randomized as well (left side to be stimulated first: n = 18; right side to be stimulated first: n = 19).

### Evaluation of migraine

For this study we applied the German version of the headache questionnaire modified according to the ICHD (3^rd^ edition)^66–68^, the headache diary of the DMKG^69^, and the MIDAS questionnaire^70,71^ (Fig. 3).

Initially, all subjects had to fill in the German version of the headache questionnaire modified according to the ICHD (3^rd^ edition) to verify migraine diagnosis by the following items: localization, duration and quality of pain, nausea, photophobia, phonophobia, and the influence of physical activity on the intensity of pain. A minimum of the mentioned criteria had to be fulfilled to receive a migraine-positive result^67,68^. Moreover, the presence of aura symptoms and an association with tension-type-headache (TTH) were recorded as well. The sensitivity and specificity of the German version of the headache questionnaire is 73% and 96% for the diagnosis of migraine, 85% and 98% for the diagnosis of TTH, and 62% and 97% for the diagnosis of a combination of both headache disorders^67^. Furthermore, the questionnaire was confirmed and revalidated to be used in epidemiological studies in order to assess the prevalence of different headache disorders^68^.

To monitor the headache frequency and characteristics the 90 days before the first rPMS session, the headache calendar of the DMKG needed to be filled in on a daily basis. With the help of the headache calendar, subjects recorded numerous items of each headache attack like date, trigger mechanisms (stress, relaxation, disturbance of sleep-awake rhythm, menstruation etc.), intensity, duration, quality, localization, forerunning symptoms (scintillating scotoma, paresthesia, aphasia etc.), concomitant symptoms like nausea, vomiting, photophobia, phonophobia or osmophobia, drug intake, dosage form, and pain relief. Subsequently, they were advised to continue filling in the headache diary during the period of stimulation sessions and also during the course of the 90 days after the last rPMS intervention. A basic diagnostic headache diary, such as the DMKG headache calendar, is a well-accepted tool that can facilitate a considerably higher diagnosis rate for subjects who filled in such a calendar for one month before consulting a specialist (complete diagnosis rate: 97.7%) when compared to subjects without any documentation of headache attacks (complete diagnosis rate: 87.7%)^69^. Moreover, a headache diary is useful to increase understanding of primary headaches and to strengthen awareness for triggers and medication intake^69^.

Besides, subjects were instructed to fill in the MIDAS questionnaire to estimate the impairment by headache events in different aspects of daily life before and after the two-week interval of rPMS application. Therefore, they had to estimate the number of days of incapacity for work and housekeeping, reduced capacity for work and housekeeping as well as absence in social activities due to headache symptoms during the 90 days before and after the interval of rPMS, respectively. The MIDAS questionnaire had to be completed prior to the first rPMS intervention and again after the 90 days of completion of the headache calendar after the last rPMS intervention. The MIDAS questionnaire has shown high internal consistency and reliability and correlates well with physicians’ clinical judgements of pain, disability, and need for medical care^70,71^. Correlation of the MIDAS score to the physicians’ assessment for “need of medical care” with r = 0.69 supports the suitability of the MIDAS questionnaire in clinical practice^70^.

### Determination of myofascial trigger points

To identify mTrPs in trapezius or deltoid muscles, a certified physiotherapist qualified for mTrP palpation examined all participants by manual examinations few days before the first stimulation session (Fig. 3). The three standard criteria defining active mTrPs were carefully checked during examination by the physiotherapist: (1) a palpable taut band with local hypersensitivity, (2) a referred pain at the typical localization of the subject’s headache must be provoked by palpation of the mTrP, (3) a spontaneous evasive movement called “jump sign” as reaction to palpation of the mTrP^47,72–74^. However, a latent mTrP does not show any referred pain recognized as the typical headache during palpation, but fulfills the following two criteria of (1) a taut band with a sensitive spot, and (2) the so-called “jump sign”^75^.

The subjects needed to show either two active mTrPs in the trapezius muscles, e.g. one active mTrP in each of them, or, alternatively, one active and one latent mTrP in the trapezius muscles. Concerning the deltoid muscles, one latent mTrP needed to be identified by the physiotherapist bilaterally. In case that more than one active or latent mTrP could be identified in one muscle, the point which was most responsive in terms of painful sensation due to manual palpation was chosen by the physiotherapist, the other points were not further considered in the study. Overall, our aim was to identify four mTrPs in each subject, one mTrP within each side of the trapezius and deltoid muscles, respectively.

The four defined mTrPs were documented by marking the chosen points with a waterproof pen. The distances between the seventh cervical vertebra and the acromion were taken as well as photos to guarantee thorough documentation of mTrP locations in each subject.

### Repetitive peripheral magnetic stimulation

During a two-week intervention period a total of six sessions of rPMS were applied to mTrPs of the trapezius or deltoid muscles, depending on group assignment, with the starting side of rPMS being alternated from session to session (Fig. 3). For stimulation, a Nexstim eXimia NBS system with a figure-of-eight stimulation coil was used (version 4.3; Nexstim Plc., Helsinki, Finland).

**Figure 4:**
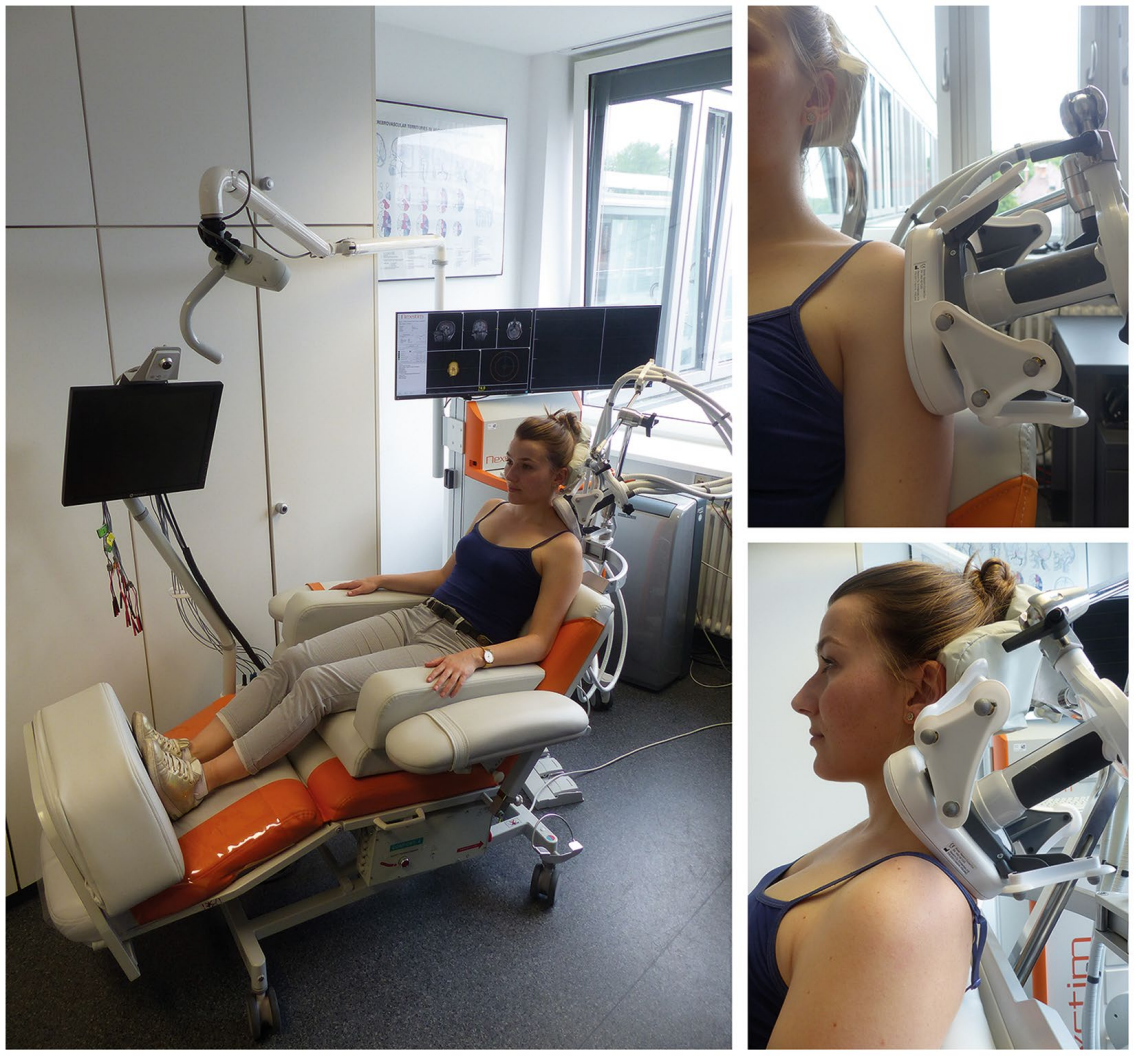
Setup of stimulation. This figure depicts the setup of stimulation by repetitive peripheral magnetic stimulation (rPMS). During pulse application to either myofascial trigger points (mTrPs) in the trapezius or deltoid muscles, the subject sat on a comfortable chair with armrests, headrest, and footplate in a relaxing position. After careful positioning of the stimulation coil over the individually defined mTrPs, a static coil holder was used to fix the correct position. Written informed consent was obtained from the subject of this figure to use this photo for publication.

At the beginning of the first rPMS session, the intensity of rPMS was defined individually on the muscles to be stimulated and was kept for both sides for the following sessions. Stimulation was initiated with an intensity of 15% of the maximum output and gradually increased by steps of 5%. The participant was advised to evaluate the sensation caused by rPMS on a visual analogue scale (VAS) from 0 (maximum comfort) to 10 (maximum discomfort and pain). We chose the highest intensity still being rated lower than 5 points on this scale for stimulation of both sides. Then, for application of a standardized stimulation protocol during each session, we fixed the stimulation coil with direct skin contact above the mTrPs of the trapezius or deltoid muscles and ensured a constant and stable position of the coil in each session (Fig. 4)^44,45^. During each visit the left and right mTrPs of the trapezius muscles (trapezius group) or the left and right mTrPs of the deltoid muscles (deltoid group) were stimulated for 15 minutes per side. Stimulation of each side consisted of 20 bursts with a total of 6,000 stimuli and a 20-Hz frequency. A single burst was composed of 300 stimuli taking 15 seconds, followed by a relaxation time of 30 seconds. Besides, there was a break of approximately two minutes between stimulation to each side, allowing the operator to change the coil position for stimulation of the contralateral side.

### Data analysis and statistics

All statistical data analyses were performed using R software (version 3.1.0; The R Foundation for Statistical Computing, Vienna, Austria) and GraphPad Prism (version 6.04; GraphPad Software Inc., La Jolla, CA, USA).

For demographics, scores of the DMKG headache calendar, and MIDAS questionnaire, descriptive statistics including mean, standard deviation (SD), median, and ranges or absolute and relative frequencies were calculated. Furthermore, the overall MIDAS score (0–5 points: none to minimal impairment, 6–10 points: mild impairment, 11–20 points: moderate impairment, and >20 points: severe impairment) was calculated based on the results of the questionnaire. This was performed separately for the trapezius group and deltoid group and separately for the pre- and post-interventional assessments, respectively. To compare demographic data and scores between subjects assigned to the trapezius or deltoid group or between pre- and post-interventional status, we used Chi-squared tests or Wilcoxon signed-rank tests. For continuous variables, non-parametric tests were performed as normal distributions could not be assumed (based on Shapiro-Wilk tests and graphical examinations). A sensitivity analysis including only female patients was performed in addition, thus excluding the single male subject included in this study (results shown in Supplementary Table 1). Correction for multiple testing was performed using the Benjamini-Hochberg procedure with a false discovery rate (FDR) of 10%^76^. The level of statistical significance was set at p < 0.05.

## Supplementary information

Supplementary Information.

## Data Availability

The datasets generated during and/or analyzed during the current study are available from the corresponding author on reasonable request.
