# Supplementary material for: Alleviation of migraine symptoms by application of repetitive peripheral magnetic stimulation to myofascial trigger points of neck and shoulder muscles – A randomized trial
Source: Sci Rep. 2020 Apr 6;10:5954. doi: 10.1038/s41598-020-62701-9 (PMC7136237; doi:10.1038/s41598-020-62701-9)
Supplement: Supplementary file 1 — Supplementary Information. [file 41598_2020_62701_MOESM1_ESM.docx]

**Alleviation of migraine symptoms by application of repetitive peripheral magnetic stimulation to myofascial trigger points of neck and shoulder muscles – A randomized trial**

Tabea Renner**^1,*^**; Nico Sollmann, MD, PhD**^2,3,*^**; Florian Heinen, MD**^1^**;

Lucia Albers, PhD**^1,4^**; Florian Trepte-Freisleder**^1^**; Birgit Klose^1^;

Helene König, MSc^1^; Sandro M. Krieg, MD, MBA**^4^**;

Michaela V. Bonfert, MD**^1,+^**; Mirjam N. Landgraf, MD**^1,+^**

*: these authors contributed equally

**^+^**: these authors contributed equally

**Supplementary Table 1:** Pre- versus post-interventional results according to the headache diary of the German Migraine and Headache Society (DMKG) and Migraine Disability Assessment (MIDAS) questionnaire in 36 female subjects (excluding one male subject)

|  | **Trapezius group**  **N=19** | | | | **Deltoid group**  **N=17** | | | |
| --- | --- | --- | --- | --- | --- | --- | --- | --- |
|  | **Pre-stimulation** | **Post-stimulation** | **Pre-Post difference** | **p*** | **Pre-**  **stimulation** | **Post-stimulation** | **Pre-Post difference** | **p*** |
|  | **Median (range)** | | |  | **Median (range)** | | |  |
| **Headache diary of the DMKG (assessed daily over the course of 90 days before and after intervention)** | | | | | | | | |
| Number of days with headache | 23 (17 - 37) | 16 (5 - 31) | 8 (-9 - 23) | **0.005** | 20 (15 - 40) | 14 (6 - 30) | 7 (-6 - 12) | **0.004** |
| Cumulative headache duration (hours) | 194 (78 - 429) | 146.5 (40 - 336) | 45 (-69 - 228) | 0.068 | 122 (60 - 482) | 102 (19 - 420) | 47.1 (-13 - 138) | 0.088 |
| Duration per headache attack (hours) | 6.8 (4.0 - 14.8) | 7.8 (3.6 - 16.9) | -0.1 (-6.6 – 4.8) | 0.606 | 5.81 (3.33-19.28) | 6.8 (2.8 - 17.5) | 0.38 (-2.10 - 7.26) | 0.617 |
| Average headache intensity (according to VAS) | 5.3 (3.5 - 6.9) | 5.9 (4.3 - 7.9) | -0.2 (-3.0 – 0.6) | 0.161 | 5.18 (3.9 - 6.53) | 5.17 (3.27-6.71) | 0.21 (-1.07 - 2.41) | 0.586 |
| Vomiting (incidences per 90 days) | 0 (0 - 4) | 0 (0 - 3) | 0 (0 – 2) | 0.523 | 0 (0 - 9) | 0 (0 - 2) | 0 (-2 - 7) | 0.824 |
| Nausea (incidences per 90 days) | 7 (0 - 25) | 4 (0 - 29) | 4 (-15 – 11) | 0.138 | 5 (0 - 16) | 4 (0 - 17) | 0 (-6 - 9) | 0.627 |
| Medication (intake per 90 days) | 12 (0 - 29) | 9 (0 - 27) | 2 (-8 – 11) | 0.254 | 11 (3 - 27) | 9 (2 - 17) | 2 (-12 - 11) | 0.427 |
| **MIDAS questionnaire (assessed for the 90 days before and after intervention)** | | | | | | | | |
| Missing school/work (days) | 1 (0 - 5) | 1 (0 - 5) | 0 (-5 - 4) | 0.914 | 1 (0 - 12) | 1 (0 - 6) | 0 (-3 - 7) | 0.842 |
| Productivity at school/work reduced by half (days) | 10 (2 - 20) | 4 (0 - 10) | 4 (-1 - 18) | **0.001** | 8 (3 - 23) | 4 (0 - 12) | 4 (-1 - 11) | **0.008** |
| Could not do household work (days) | 5 (0 - 11) | 2 (0 - 15) | 1 (-5 - 9) | 0.095 | 5 (0 - 18) | 2 (0 - 12) | 2 (-8 - 14) | 0.158 |
| Household work productivity reduced by half (days) | 5 (0 - 15) | 2 (0 - 7) | 3 (-3 - 14) | **0.002** | 7 (0 - 14) | 3 (0 - 11) | 3 (-11 - 12) | 0.095 |
| Missing family, social or leisure activities (days) | 3 (0 - 10) | 2 (0 - 10) | 1 (-4 - 4) | 0.324 | 5 (0 - 17) | 2.5 (0 - 12) | 2 (-5 - 11) | 0.177 |

* Wilcoxon signed rank test. P-values printed in bold are statistically significant after correction for multiple testing using the Benjamini-Hochberg procedure with a false discovery rate (FDR) of 10%.
